# Rally at Houston

**Published:** 1905-03

**Authors:** 


### THE TEXAS MEDICAL JOURNAL.

AUSTIN, TEXAS.

A MONTHLY JOURNAL OF MEDICINE AND SURGERY.

EDITED AND PUBLISHED BY
F. E. DANIEL, M. D.
Mrs. F. E. DANIEL, Business Manager.

Published Monthly at Austin, Texas. Subscription price $1.00 a year in advance.

Eastern Representative: John Guy Monihan, St. Paul Building, 220 Broadway New York City.

Official organ of the West Texas Medical Association, the Houston District Medical Association, the Austin District Medical Society, the Brazos Valley Medical Association, the Galveston County Medical Society, and several others.

### RALLY AT HOUSTON.

The medical profession of the State is now pretty thoroughly organized. It would seem to be in position to make its influences felt in the interest of the public health and safety, yet the legislation asked for at the present session of the Legislature (an amendment to the existing Medical Practice Act) was "turned down cold" by the Senate committee (Judiciary No. 2—Davidson, chairman). This bill was prepared by the State Board of Medical Examiners; discussed by the full House of Delegates, and unanimously agreed to. The Committee on Public Policy and Legislation, who were entrusted with the duty of securing its passage by the Legislature, were a unit on the necessity of such amendment. It was ratified a second time by the House of Delegates in called session. It thus represented the opinion practically of the entire medical profession of the State that the public need protection from ignorant "doctors" who, under the law, have license to practice without "license" and without examination, and that the proposed plan was the best means of securing that protection. This advice and opinion was ignored, and the bill killed in committee. Senators Looney and McKamy were appointed to draft a bill as a substitute for it, and the substitute provided for a mixed board, with Osteopaths, Physiomedicals, Homeopaths, and Eclectics on it, one each, and five physicians.

Chairman Davidson, who so ably championed a similar bill last session, and who had been selected by the committee to introduce and champion it, disappointed us greatly. He was unfortunately absent almost continuously the first six weeks of the session, and absent on the 7th of February, when his vote would have tied, and his influence defeated the Osteopath board bill. He refused to support our bill before the committee, and refused to let it be withdrawn by the committee. He took offense because, as a last dispairing effort to save the bill (he always said it would not pass, anyhow), Drs. Daniel and Wooten, of the committee, accepted an amendment which relieved Christian Scientists of examination in medicine provided they were not allowed to undertake surgery, contagious diseases, and obstetrics. This was not only just, as I have always contended, because to require them to be examined in anatomy and other branches, would be tantamount to prohibition altogether, and public sentiment and sympathy would never consent to a total crushing out by law of this cult; but it was advocated as expedient. Granting this very reasonable contention, the Christian Scientists were disarmed, and would become our allies. So, Mr. Davidson killed the bill by saying that he would not support it. Indeed, from the two brief interviews I had with him, it seemed to me that he did not want to support it; feeling that, as he said, it would not pass, he did not want to be on the losing side. Senator Hicks, who had actively supported him and us last session, had become convinced since last session that 125 Osteopaths are *right*, and the entire medical profession of the State are *wrong*, and I assume that, naturally, he did not wish to cross swords with his quondam protagonist. It is most remarkable, the persuasive eloquence of these one hundred Osteopaths! Demosthenes and Cicero are not a circumstance to them when it comes to persuasive eloquence and convincing logic. There is an overwhelming sentiment in the Legislature in favor of recognizing every sect in medicine and giving them authority to license themselves. It is a condition that strikes at the vitals of legitimate medicine, and if it is not checked and the people educated to see the folly of it, the usefulness of our association is forever destroyed.

Hence, I urge every member who possibly can do so, to attend the meeting at Houston, April 25-28. The situation calls for the best thought and mature deliberate judgment. Let us see if we can develop wisdom enough and influence enough by next session to avert the pending disgrace to the State of giving every "pathy" recognition and practically exemption from the law. We must be ready for the Thirtieth. It is not likely that anything will be done in the way of boards this session.

